# The Relationship Between Attention and Suicidal Ideation Among Patients With Adult‐Onset Chronic Schizophrenia

**DOI:** 10.1002/brb3.70079

**Published:** 2024-10-08

**Authors:** Qiongzhang Wang, Yurou Zhou, Weiqian Xu, Wei Tang, Jing Liu

**Affiliations:** ^1^ Department of Neurology The First Affiliated Hospital of Wenzhou Medical University Wenzhou Zhejiang China; ^2^ The First School of Medicine Wenzhou Medical University Wenzhou Zhejiang China; ^3^ Taizhou Second People's Hospital Taizhou Zhejiang China; ^4^ Zhejiang Provincial Clinical Research Center for Mental Disorders The Affiliated Kangning Hospital of Wenzhou Medical University Wenzhou Zhejiang China

**Keywords:** adult‐onset, cognitive impairment, positive symptoms, schizophrenia, suicidal ideation

## Abstract

**Background:**

Patients with chronic schizophrenia (SZ) have a high risk of suicide, and their cognition function is impaired. We aimed to explore the relationship between attention and suicidal ideation among patients with adult‐onset chronic SZ.

**Methods:**

A total of 416 patients with adult‐onset chronic SZ were enrolled in this study. We divided patients into suicidal ideation group and non‐suicidal ideation group according to the evaluation results of the Beck Scale for Suicide Ideation. Psychotic symptoms were measured by Positive and Negative Syndrome Scale (PANSS), and cognitive function was measured by Repeatable Battery for the Assessment of Neuropsychological Status (RBANS). Insomnia symptoms were measured by Insomnia Severity Index (ISI).

**Results:**

Age was significantly different (44.28 ± 10.58 vs. 48.46 ± 12.23, *t* = 10.64, *p* = 0.001) between the two groups, and the patients with suicidal ideation were younger than patients without suicidal ideation. The positive symptom scores of the PANSS, the scores of ISI, and attention scores of RBANS were higher in patients with suicidal ideation than patients without suicidal ideation (17.30 ± 5.67 vs. 15.58 ± 4.90, *t* = 9.633, *p* = 0.002; 3.00 [1.00–6.00] vs. 2.00 [1.00–3.50], *Z* = −2.048, *p* = 0.041; 81.80 ± 14.99 vs. 76.91 ± 13.88, *t* = 10.101, *p* = 0.002). In the logistic regression analysis, age (odds ratio [OR], 0.973; 95% confidence interval [95%CI], [0.955–0.992]; *p* = 0.005), positive symptom scores of PANSS (OR, 1.063; 95%CI, [1.019–1.109]; *p* = 0.005), ISI scores (OR, 1.098; 95%CI, [1.037–1.163]; *p* = 0.001), and attention scores of RBANS (OR, 1.029; 95%CI, [1.013–1.047]; *p* = 0.001) were independently associated with the occurrence of suicidal ideation among patients with adult‐onset chronic SZ.

**Conclusions:**

High attention scores of RBANS were a risk factor for suicidal ideation among patients with adult‐onset chronic SZ.

## Graphical abstract

To the best of our knowledge, this is the first study to detect the relationship between distinct cognitive domains and suicidal ideation among patients with adult‐onset chronic SZ. High attention scores of RBANS were a risk factor for suicidal ideation among patients with adult‐onset chronic schizophrenia. It might help us clarify the underlying mechanism of suicidal ideation and provide a new proposal for reducing the occurrence of suicidal ideation.

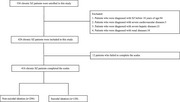

## 1 Introduction

Schizophrenia (SZ) is a common mental disorder, which has a prevalence of approximately 0.28% (Charlson et al. 2018). Chronic SZ accounts for a large proportion of SZ (Ruiz‐Iriondo et al. 2019). In addition, more than two‐thirds of SZ are adult‐onset SZ (Remschmidt and Theisen 2012), which accounts for a large proportion of SZ. SZ patients have a higher rate of suicide than the general population (Bareis et al. 2023). The prevalence of suicide in SZ patients ranges from 10% to 50% (Ventriglio et al. 2016). It is estimated that SZ will shorten life expectancy by 10 to 20 years, and suicide is the biggest cause of shortening life expectancy (Balhara and Verma 2012; Lopez‐Morinigo et al. 2016). Suicidal ideation has been proved to be a significant predictor of suicide attempt (Galfalvy et al. 2023). Suicidal ideation in SZ patients leads to 20‐fold greater suicide rates than those in the general population (Andriopoulos et al. 2011). Suicidal ideation harms the patient's quality of life (Pelizza et al. 2020). Given that China has the largest number of SZ patients in the world, how to properly treat and care for these patients with suicidal ideation is meaningful.

Suicidality is a complex social phenomenon, which is involved in sociological, psychological, and biological factors. Many studies have explored the risk factors of suicidal ideation among patients with SZ, and many risk factors have been found to be associated with suicidal ideation of SZ. These risk factors included demographic variables (such as younger age, male, and higher premorbid IQ (Cassidy et al. 2018)), clinical characteristics (such as chronic disease courses, younger age at onset, longer duration of untreated psychosis, increased positive symptoms, decreased negative symptoms (Chong et al. 2020), insomnia symptoms (Ketcham et al. 2024), a greater level of insight (Flanagan and Compton 2012), and fewer cognitive deficits (Villa et al. 2018)), sociological factors (such as poor social function and poor social support (Pompili et al. 2007; Silva et al. 2018)), and psychological factors (such as emotional problems, hopelessness (Pompili et al. 2007), and psychological pain (Bohaterewicz et al. 2021)).

Previous studies have reported that preserved neurocognitive functions are related to suicidality in SZ patients (Delaney et al. 2012). Unlike sociological risk factors and psychosocial risk factors, preserved cognition function is necessary in the process of suicidal ideation and suicidal behavior. Some researchers have suggested that patients with awareness of illness are at greater risk for suicidal behavior (Amador et al. 1996). Such individuals are more likely to develop a sense of hopelessness and demoralization, which leads to suicidal behavior. In addition, preservation of higher executive function may influence the ability to initiate and plan suicidal behavior (Nangle et al. 2006). This may explain why there is a correlation between neurocognition and suicidality in SZ patients. Neurocognition is a basic function of the central nervous system, including a multitude of cognitive domains such as executive function, working memory, process speed, attention, and episodic memory (Sheffield and Barch 2016). The associations between distinct neurocognitive domains and suicidality in SZ patients have been largely mixed (Delaney et al. 2012; Huang et al. 2021; Zhang et al. 2018). A study carried on outpatients with SZ has shown that attention, memory, and verbal fluency are associated with suicidal ideation (Villa et al. 2018). Delaney et al. (2012) have reported that attention and memory are associated with suicidal attempt. Zhang et al. (2018) have found that only attention is associated with suicidal attempt in first‐episode and drug‐naive patients with SZ. As an important component of executive function and insight, attention might be a vital cognitive domain for suicidality. Few studies have focused on the relationship between attention and suicidal ideation in SZ inpatients. Meanwhile, no study has been done to explore the relationship between attention and suicidal ideation in adult‐onset chronic SZ patients.

Given that there is no study focused on the relationships between attention and suicidal ideation among the adult‐onset chronic SZ patients, we aimed to explore the relationship between attention and suicidal ideation among patients with adult‐onset chronic SZ. It might help us clarify the underlying mechanism of suicidal ideation and provide a new proposal for reducing the occurrence of suicidal ideation.

## 2 Materials and Methods

### 2.1 Participants

This is a cross‐sectional analytical and descriptive study. Patients who were diagnosed with adult‐onset chronic SZ by psychiatrists at the Affiliated Kangning Hospital of Wenzhou Medical University were included in our study from December 2018 to December 2019. All patients were assessed by Structured Clinical Interview for DSM‐IV (SCID). All patients were inpatients. The clinical research described in the manuscript was carried out in accordance with the Declaration of Helsinki. All patients met the inclusion criteria are as follows: (1) A diagnosis of SZ according to the Diagnostic and Statistical Manual of Mental Disorders, Fourth Edition (DSM‐IV), (2) age 18–70 years, (3) diagnosed with SZ in adulthood (>18 years old), (4) duration of SZ is more than 5 years and defined as chronic SZ, (5) at stable phase of SZ, (6) Han Chinese ethnicity. The exclusion criteria as follows: (1) Patients with a concomitant physical disease severe enough that they could not complete the measurements (e.g., cardiovascular, liver, kidney, gastrointestinal diseases, epilepsy, or head injury), (2) the female patients who are pregnant, planning to become pregnant, or breastfeeding during the study period, (3) with a history of alcohol or other substance abuse or dependence.

### 2.2 Clinical Measurements

A complete survey in this study was performed using a case report form (CRF). When patients were included, demographic and clinical data (age, gender, years of education, marital status [married, divorced, or widowed], current smoking status, and body mass index [BMI]) were recorded. Family history of mental disease, age at onset, duration of SZ, and psychiatric drug equivalent dose were also recorded on the CRF.

### 2.3 Psychological Assessment

#### 2.3.1 Assessment of Suicidal Ideation

The Beck Scale for Suicide Ideation‐Chinese Version (BSI‐CV) (www.crisis.org.cn) was used to evaluate the current intensity of suicidal ideation, which was defined as thoughts about ending one's own life (whether active [with a plan] or passive [with only a wish to die but no specific plan]) (Turecki et al. 2019) among the patients with SZ (Beck, Steer, and Ranieri 1988). BSI‐CV used 19 items to evaluate suicidal ideation in the past week. The first five items were screening items. Only when the answer to Item 4 (active suicidal ideation) or Item 5 (passive suicidal ideation) was “weak” or “moderately to strong” (i.e., it was not 0), the subjects will continue to complete the next Items 6–19. Otherwise, the scale survey was ended. On the basis of their scores for Items 4 or 5 on the Beck Scale for Suicide Ideation, all chronic SZ patients were divided into the SZ with suicidal ideation group and the SZ without suicidal ideation group. A score for Item 4 (desire to make an active suicidal attempt) or Item 5 (passive suicidal desire) of “weak” or “moderate to strong” indicated patients with suicidal ideation, and only when the scores of Items 4 and 5 were definitely “not” indicated patients without suicidal ideation (Boudreaux et al. 2017; Liu et al. 2023).

#### 2.3.2 Assessment of Clinical Variables and Cognitive Function

Positive and Negative Syndrome Scale (PANSS) was used to evaluate the severity of psychotic symptoms in SZ patients (Kay, Fiszbein, and Opler 1987), which is consisted of three domains: positive, negative, and general symptoms. The Repeatable Battery for the Assessment of Neuropsychological Status (RBANS), including attention, speech, visual span, immediate memory, and delayed memory dimensions, was used to evaluate cognitive function in this study (Randolph et al. 1998). We used the translated Chinese version of this test, which has established validity and test–retest reliability among patients with SZ and healthy controls (Hui et al. 2016). In the current study, Cronbach's alpha of the RBANS was 0.770. The Insomnia Severity Index (ISI) was used to evaluate the severity of insomnia symptoms (Bastien, Vallieres, and Morin 2001). In the current study, Cronbach's alpha of the ISI was 0.874. All treatment information was reviewed and confirmed by experienced psychiatrists. All these scales have been widely used to assess clinical symptoms in SZ patients. All assessments were performed independently by two trained psychiatrists with a correlation coefficient greater than 0.8.

### 2.4 Statistical Analysis

The results were presented as the means ± standard deviations (SDs) or medians (interquartile ranges) for continuous variables depending on the normal or non‐normal distribution of data, whereas categorical variables were expressed as numbers (percentages). Student's *t* test was used to explain the differences between the normally distributed variables, whereas the Mann–Whitney *U* test was used to the non‐normal distributed variables. The categorical variables were compared using the chi‐squared test. The roles of cognitive function in suicidal ideation were checked by stepwise multivariate logistic regression analysis (forward: conditional model) after adjusting for all potential risk factors related to suicidal ideation in univariate analysis. All statistical tests were performed with SPSS for Windows (Release 22.0; SPSS, Chicago, IL, USA). Values of *p* < 0.05 were considered to be statistically significant in all tests.

## 3 Results

### 3.1 Demographic Characteristics

**FIGURE 1:**
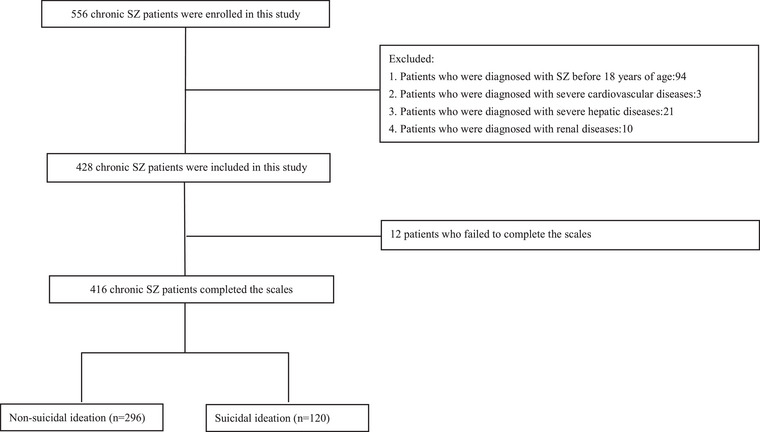
A total of 556 patients with chronic schizophrenia were screened, and 428 patients were included in this study. There were 12 (2.8%) patients failed to complete the scales, and 416 (97.2%) patients completed the scales in our study. The remaining 416 patients were divided into the non‐suicidal ideation group (*n* = 296) and the suicidal ideation group (*n* = 120) on the grounds of the absence or presence of suicidal ideation.

A total of 556 patients with chronic SZ were screened, and 428 patients were included in this study. There were 12 (2.8%) patients failed to complete the scales, and 416 patients completed the scales in our study (Figure 1). The response rate to the scale was 97.2%. A total of 416 patients with chronic SZ were enrolled in our study, which was sufficient after evaluation by G‐Power. The G‐Power of this study was 0.996. Among 416 patients, the average age of patients with adult‐onset chronic SZ was 47.25 ± 11.99 years old, their average years of education were 9.30 ± 3.10 years, and their average BMI was 24.78 ± 4.17. The median age at onset was 25 years, and the low and upper quartiles were 22 and 30 years, respectively. The median duration of SZ was 20 years, and the low and upper quartiles were 11 and 30, respectively. The prevalence of suicidal ideation reached 28.85% in this study, which represented no significant difference in sex distribution. There was a negative correlation between the duration of SZ and attention scores of RBANS (*r* = −0.099, *p* = 0.043). Meanwhile, there was a negative correlation between the duration of SZ and immediate memory scores of RBANS (*r* = −0.125, *p* = 0.011). There was no correlation between the duration of SZ and the total scores of RBANS (*r* = −0.051, *p* = 0.295). No correlation was found between the duration of SZ and other RBANS subcategories (all *p* > 0.05).

The SZ patients with suicidal ideation were younger (44.28 ± 10.58 vs. 48.46 ± 12.23, *t* = 10.64, *p* = 0.001), had a shorter duration of illness at the time of assessment (16.50 [10.25–27.75] vs. 21.00 [12.00–30.00], *Z* = −2.141, *p* = 0.032), and had a higher BMI (25.47 ± 4.49 vs. 24.50 ± 4.01, *t* = 4.650, *p* = 0.032) than the patients without suicidal ideation. More details on the demographic information results could be found in Table 1.

**TABLE 1:** Social‐demographics in adult‐onset chronic schizophrenia patients with and without suicide ideation.

	Non‐suicidal ideation (*n* = 296)	Suicidal ideation (*n* = 120)	*t*/*Z*/*χ* ^2^	*p*
Gender (female percentage)	88.00 (29.73)	44.00 (36.67)	1.897	0.168
Age (years)	48.46 ± 12.23	44.28 ± 10.58	10.64	0.001**
Education year (years)	8.00 (8.00–11.00)	9.00 (8.00–12.00)	− 1.457	0.145
Marriage status, *n* (%)			2.053	0.562
Unmarried	171.00 (57.78)	69.00 (57.50)		
Married	66.00 (22.29)	23.00 (19.17)		
Divorced	53.00 (17.90)	27.00 (22.50)		
Widowed	6.00 (2.03)	1.00 (0.83)		
Current smoking, *n* (%)	90.00 (30.41)	34.00 (28.33)	0.175	0.626
BMI (kg/m^2^)	24.50 ± 4.01	25.47 ± 4.49	4.650	0.032*
Family history of mental diseases, *n* (%)	43.00 (14.53)	23.00 (19.17)	1.377	0.241
Age of onset (years)	25.00 (22.00–31.00)	25.00 (21.25–28.00)	− 1.251	0.211
Duration of schizophrenia (years)	21.00 (12.00–30.00)	16.50 (10.25–27.75)	− 2.141	0.032*
Psychiatric drug equivalent dose (CPZ equivalent mg)	240.00 (146.25–363.75)	272.50 (137.50–417.50)	− 0.788	0.431

Abbreviations: BMI, body mass index; CPZ, chlorpromazine.

*p < 0.05.

**p < 0.01.

### 3.2 Psychotic Symptoms, Cognitive Function, and Insomnia Symptoms in Patients With or Without Suicidal Ideation

The positive symptom scores of PANSS were significantly higher in the suicidal ideation group than the non‐suicidal ideation group (17.30 ± 5.67 vs. 15.58 ± 4.90, *t* = 9.633, *p* = 0.002). Suicidal ideation patients had higher total scores of RBANS (68.25 ± 13.97 vs. 63.88 ± 11.58, *t* = 10.780, *p* = 0.001) than patients without suicidal ideation, specifically for immediate memory (58.76 ± 16.12 vs. 54.32 ± 12.84, *t* = 8.749, *p* = 0.003), visuospatial scores (82.73 ± 19.43 vs. 78.28 ± 16.95, *t* = 5.387, *p* = 0.021), and attention (81.80 ± 14.99 vs. 76.91 ± 13.88, *t* = 10.101, *p* = 0.002). Patients with suicidal ideation had higher scores of ISI than patients without suicidal ideation (3.00 [1.00–6.00] vs. 2.00 [1.00–3.50], *Z* = −2.048, *p* = 0.041). Consequently, patients with suicidal ideation were more likely to be young, had a high BMI, and had a short duration of SZ. Meanwhile, they had severe positive symptoms, less cognitive impairment, and severe insomnia symptoms. More details could be found in Table 2.

**TABLE 2:** Clinical characteristics in adult‐onset chronic schizophrenia patients with and without suicide ideation.

	Non‐suicidal ideation (*n* = 296)	Suicidal ideation(*n* = 120)	*t*/*Z*	*p*
PANSS				
Positive symptoms scores	15.58 ± 4.90	17.30 ± 5.67	9.633	0.002**
Negative symptoms scores	20.90 ± 6.24	20.09 ± 6.52	1.390	0.239
General symptoms scores	38.85 ± 7.89	40.29 ± 8.60	2.698	0.101
The total scores	75.33 ± 15.70	77.68 ± 17.29	1.811	0.179
RBANS				
Immediate memory scores	54.32 ± 12.84	58.76 ± 16.12	8.749	0.003**
Visuospatial scores	78.28 ± 16.95	82.73 ± 19.43	5.387	0.021*
Language scores	79.65 ± 13.21	80.55 ± 12.59	0.411	0.522
Attention scores	76.91 ± 13.88	81.80 ± 14.99	10.101	0.002**
Delayed memory scores	60.00 (48.00–80.00)	68.00 (44.00–83.00)	− 1.444	0.149
The total scores	63.88 ± 11.58	68.25 ± 13.97	10.780	0.001**
ISI	2.00 (1.00–3.50)	3.00 (1.00–6.00)	− 2.048	0.041*

Abbreviations: PANSS, Positive and Negative Syndrome Scale; RBANS, Repeatable Battery for the Assessment of Neuropsychological Status; ISI, Insomnia Severity Index.

*p < 0.05.

**p < 0.01.

### 3.3 Logistic Regression of Risk Factors for Suicidal Ideation

Logistic regression analysis was applied to investigate the risk factors of suicidal ideation among the patients with adult‐onset chronic SZ. The role of attention in suicidal ideation was evaluated by logistic regression analysis after adjusting for all potential risk factors related to suicidal ideation in univariate analysis. The results demonstrated that age (odds ratio [OR], 0.973; 95% confidence interval (95%CI), [0.955–0.992]; *p* = 0.005), positive symptoms scores of PANSS (OR, 1.063; 95%CI, [1.019–1.109]; *p* = 0.005), ISI (OR, 1.098; 95%CI, [1.037–1.163]; *p* = 0.001) scores, and attention scores of RBANS (OR, 1.029; 95%CI, [1.013–1.047]; *p* = 0.001) were associated with suicidal ideation. More details could be found in Table 3.

**TABLE 3:** The risk factors for suicidal ideation in adult‐onset chronic schizophrenic patients.

	OR	95%CI	*p*
Age	0.973	0.955–0.992	0.005**
Positive symptoms scores	1.063	1.019–1.109	0.005**
Attention scores	1.029	1.013–1.047	0.001**
ISI	1.098	1.037–1.163	0.001**

Abbreviations: CI, confidence interval; ISI, Insomnia Severity Index; OR, odds ratio.

*p < 0.05.

**p < 0.01.

## 4 Discussion

To the best of our knowledge, this is the first study to detect the relationship between distinct cognitive domains and suicidal ideation among patients with adult‐onset chronic SZ. We found that high attention scores of RBANS were a risk factor for suicidal ideation among patients with adult‐onset chronic SZ, which might provide a new and extra proposal for reducing the occurrence of suicide. The results of this study were as follows: (1) A prevalence of suicidal ideation was 28.85% among the inpatients with adult‐onset chronic SZ. (2) Suicidal ideation patients were younger than non‐suicidal ideation patients. (3) Suicidal ideation patients had suffered more positive symptoms than non‐suicidal ideation patients. (4) Suicidal ideation patients had more insomnia symptoms than non‐suicidal ideation patients. (5) Suicidal ideation patients had higher attention scores of RBANS than non‐suicidal ideation patients.

In this study, we found that 28.85% of patients with adult‐onset SZ had suicidal ideation, which is consistent with the results of previous studies carried out among patients with SZ (Fenton et al. 1997; Minzenberg et al. 2014; Radomsky et al. 1999; Ran et al. 2004). Moreover, we found that suicidal ideation patients were younger than non‐suicidal ideation patients among adult‐onset chronic SZ patients (Kasckow, Golshan, and Zisook 2014). Many previous studies carried out among SZ patients have reported that young patients have high prevalence of suicide, which is similar with our results (Okusaga et al. 2021; Olfson et al. 2021). However, another study carried on middle aged and older patients with SZ has announced that there is no association between age and suicidal ideation (Kasckow, Golshan, and Zisook 2014). This difference in the results might be explained by the age difference of patients with SZ in the two studies. A positive effect of age on mental health‐related quality of life (HRQOL) has been found in the previous study (Folsom et al. 2009). The old patients with SZ perceive and adjust to their illnesses well (Shepherd et al. 2012), which might be used to explain the low prevalence of suicidal ideation among the old patients. Although the duration of SZ cannot be an independent variable in the logistics regression, the statistical difference in Table 2 can also illustrate that the patients with short duration of SZ seem to be likely to have a suicidal ideation. It might be explained by the good adaptation to living with some stable symptoms among the patients with long duration of SZ (Shepherd et al. 2012).

Severe positive symptoms were proved to be a risk factor for suicidal ideation in our study, which is consistent with previous studies (Kjelby et al. 2015; Yan et al. 2013). A study conducted in China also has demonstrated that patients with suicidal ideation suffer more severe delusions and hallucinations (Xiang et al. 2008). We speculated that there were two reasons. First, patients may suffer imperative auditory hallucinations or persecutory delusion. Second, a long‐term illness may make the patients feel fearful, anxious, and desperate. Meanwhile, we detected the relationship between insomnia symptoms and suicidal ideation. Higher scores of ISI were associated with suicidal ideation in adult‐onset chronic SZ patients, which is consistent with recent studies among chronic SZ patients (Miller, McCall et al. 2021; Miller, McEvoy, and McCall 2021; Miller et al. 2019). The underlying mechanism is still unclear. Several psychological and physiological mechanisms may mediate the association between insomnia and suicidal ideation in SZ such as hopelessness, serotonergic dysfunction, and hypothalamic–pituitary–adrenal (HPA) axis dysfunction (McCall and Black 2013). As we know, chronic insomnia patients are more likely to feel desperate because of the dysfunctional beliefs and attitudes about sleep (McCall and Black 2013), which may lead to suicide (Simpson et al. 2011). In addition, the sleep deprivation of mice leads to a loss of sensitivity of post‐synaptic 5‐HT receptors (Novati et al. 2008; Roman et al. 2005), and this serotonin desensitization is accompanied by a blunted HPA axis (Novati et al. 2008). Both serotonergic dysfunction and HPA axis dysfunction jointly lead to the occurrence of suicidal ideation (Pompili et al. 2010).

In this study, suicidal ideation group had better attention than non‐suicidal ideation group, which is consistent with the studies carried on major psychotic disorder patients and SZ outpatients (Nangle et al. 2006; Villa et al. 2018). The underlying mechanism may be that SZ patients with good attention tend to have good insight and execution function. Good insight and execution function have been affirmed to be associated with suicidality (Amador et al. 1996; Nangle et al. 2006; Tsuang 1978; Verma et al. 2016). Attention belongs to a domain of the RBANS and is used to measure a person's ability to selectively concentrate on a discrete aspect of information. Cognitive insight refers to the patients’ capacity to evaluate their atypical experiences and misinterpretations of events such as awareness of illness (Beck et al. 2004). The SZ patients with good attention may have better insight and awareness of their illness and its debilitating effects (Delaney et al. 2012). The SZ patients who are aware of delusions, anhedonia, blunted affect (Amador et al. 1996), the need for treatment (Schwartz 2000; Schwartz and Petersen 1999), and the social consequences of the disorder (Schwartz 2000) would be vulnerable to having suicidal ideation and suicide attempts. Executive function is the ability to shift attention from one stimulus to another, initiate or stop engaging in specific behaviors, assess risks, and develop action plans and implement them (Nangle et al. 2006). This impairment of supervisory attentional system will result in the difficulties with goal formulation and an inability to plan effectively (Nangle et al. 2006; Randolph et al. 1998). Thus, those SZ patients with relatively higher attention may have greater ability to formulate suicidal ideation and initiate suicidal attempts (Nangle et al. 2006).

There are several limitations in the study. First, this is a cross‐sectional study, which cannot show direct causality between suicidal ideation and the risk factors in patients with adult‐onset chronic SZ. Second, the all subjects were inpatients, and the selection biases may limit the generalizability of the findings. Third, we have no healthy control group. Fourth, compared with SCID, Beck SI scale is not the best way to assess the suicidal ideation of SZ patients. Fifth, considering the complexity of the neurocognition, the result measured by the RBANS may not be particularly perfect and some domains of cognition related with suicidal ideation may not be detected. Sixth, the age span of the patients in this study is large. SZ patients showed deterioration in attention with increasing age, which may lead to biased results. Hence, a cohort study is needed to confirm our results and explore whether there is an association between attention and suicidal ideation among the chronic SZ patients.

## 5 Conclusion

Our study confirmed the high prevalence of the suicidal ideation among the adult‐onset chronic SZ inpatients. The young patients with adult‐onset chronic SZ had a higher risk of suicidal ideation compared with the senior. And the adult‐onset chronic SZ patients with suicidal ideation suffered severe positive symptoms and insomnia symptoms. Meanwhile, the adult‐onset chronic SZ patients with suicidal ideation performed good attention function. Hence, we should devote additional concern on the young chronic SZ with prominent psychotic symptoms, insomnia symptoms, and good attention function. An effective intervention focusing on distracting attention from the disease to reduce the occurrence of suicidal ideation should be proposed. Further cohort studies are warranted to detect the relationship between attention function and suicidal ideation among chronic SZ patients.

## Author Contributions

**Qiongzhang Wang**: formal analysis, funding acquisition, methodology, software, visualization, writing–original draft. **Yurou Zhou**: visualization, writing–review, and editing. **Weiqian Xu**: data curation, investigation. **Wei Tang**: conceptualization, funding acquisition, supervision. **Jing Liu**: conceptualization, resources, supervision, writing–review and editing.

## Ethics Statement

This study was approved by the Medical Ethics Committee of the Affiliated Kangning Hospital of Wenzhou Medical University (Ethics Number: YJ‐2024‐34‐01).

## Consent

The informed consents were signed by the patients and their relatives.

## Conflicts of Interest

The authors declare no conflicts of interest.

### Peer Review

The peer review history for this article is available at https://publons.com/publon/10.1002/brb3.70079.

## Data Availability

The data that support the study conclusions are available from the corresponding author upon reasonable request.
